# Editorial: Clinical Cardiopulmonary Exercise Testing

**DOI:** 10.3389/fphys.2021.711505

**Published:** 2021-06-28

**Authors:** Denis E. O'Donnell, Pierantonio Laveneziana, J. Alberto Neder

**Affiliations:** ^1^Respiratory Investigation Unit and the Laboratory of Clinical Exercise Physiology, Queen's University and Kingston Health Sciences Centre, Kingston, ON, Canada; ^2^Sorbonne Université, Faculté de Médecine Pierre et Marie Curie & APHP and Service d'Explorations Fonctionnelles de la Respiration, de l'Exercice et de la Dyspnée, Hôpital Universitaire Pitié-Salpêtrière, Tenon et Saint Antoine, Paris, France

**Keywords:** cardiopulmonary exercise testing, inspiratory neural drive, dynamic respiratory mechanics, exertional dyspnea, physiological characterization

The value of cardiopulmonary exercise testing (CPET) is well-established as an adjunct to clinical evaluation. Worldwide, exercise “stress” testing is vastly under-utilized, especially for individuals with pulmonary disorders. This is lamentable because modern refinements in the measurements of physiological impairment during CPET have improved our ability to evaluate such patients more comprehensively (Domnik et al.; Phillips et al.; James et al.). Thus, the overarching objective of the current Research Topic series was to review some of these recent advancements in CPET methods and interpretation and to discuss their relevance, in both clinical and research settings, in patients with various respiratory conditions.

Uniquely, CPET can identify specific abnormalities of the integrated metabolic, cardiopulmonary, locomotor muscle, and neurosensory systems, under graded physiologic stress. Based on foundational physiological principles, CPET interpretation has traditionally focused on measuring peak oxygen uptake (*V*O_2_)—a key metric of exercise performance—and an independent predictor of mortality in various cardio-pulmonary disorders (Killian and Jones, 1984; Jones, 1997; Wasserman et al., 1999, 2012; Jones and Killian, 2000). Traditional interpretative algorithms incorporate quantitative estimates of cardiac and ventilatory reserves, as well as aerobic capacity, to help explain reduced peak *V*O_2_ in the individual. However, it must be remembered that low peak *V*O_2_ often reflects intolerable exertional symptoms (e.g., dyspnea and leg discomfort), well before physiological maxima are actually attained (Neder et al.). Thus, measurement of exertional symptoms, using validated scales, is integral to any assessment of exercise capacity (Laviolette and Laveneziana, 2014). Moreover, it is now clear that traditional estimates of breathing and cardiac reserves, based on ratios of peak ventilation and heart rate, to their respective maxima, can sometimes be misleading. Thus, apparent preservation of breathing and cardiac reserves can often obscure important respiratory mechanical constraints and severe dyspnea at relatively low exercise intensities which, in turn, explain low peak *V*O_2_ (Neder et al.).

Recent experimental work during CPET examined the simultaneous associations between inspiratory neural drive (IND) to the respiratory muscles, dynamic respiratory mechanics and subjective breathing discomfort and has shed light on fundamental mechanisms of exercise limitation in health and various respiratory conditions (Domnik et al.) (Laveneziana et al., 2009, 2011, 2013a,b, 2014, 2015; Laviolette and Laveneziana, 2014; O'Donnell et al., 2017, 2019; Neder et al., 2019; Boucly et al., 2020). In healthy participants, several acute physiological adaptations to the increasing metabolic demand of exercise, ensure admirable preservation of harmonious coupling between increasing IND and the ventilatory response of the dynamic respiratory system, such that arterial blood gas and acid-base homeostasis are achieved, and dyspnea is minimized. However, these remarkable, integrated cardio-respiratory adjustments to exercise become variably undermined by the presence of pulmonary diseases, even when resting pulmonary function appears normal. Thus, disease-related disruption of normal cardio-respiratory adaptations to exercise requires large compensatory increases in IND to maintain adequate alveolar ventilation. This, in conjunction with a widening disparity between increasing IND and the abnormal mechanical response of the impaired respiratory system (neuro-mechanical dissociation, NMD) has serious deleterious sensory consequences at higher exercise intensities, leading to premature termination of exercise (Domnik et al.).

Most chronic respiratory conditions are characterized by excessive ventilation for a given metabolic load during exercise, when compared with healthy controls. This ultimately points to increased IND due to multiple physiological perturbations, including: the effect of high physiological dead space, increased respiratory muscle loading, early lactic acidosis, and critical hypoxemia. Additionally, increased sympathetic nervous system activation and altered afferent activity from receptors in the respiratory and locomotor muscles, baroreceptors and chemoreceptors are undoubtedly contributory, but difficult to measure. High ventilatory equivalent for CO_2_ (*V*_E_/*V*CO_2_) is linked to increased IND and dyspnea intensity and is common to several respiratory conditions, including chronic obstructive pulmonary disease (COPD) (Phillips et al.), interstitial lung disease (ILD), (Molgat-Seon et al.) and pulmonary arterial hypertension (PAH) (Laveneziana and Weatherald). It is no surprise, therefore, that *V*_E_/*V*CO_2_ is increasingly represented in CPET interpretative algorithms (Phillips et al.). High *V*_E_/*V*CO_2_ nadir (e.g., > 34) generally indicates presence of poor ventilatory efficiency, due to high physiological dead space (and compromised lung CO_2_ elimination), alveolar hyperventilation (enhanced chemo-sensitivity) or both. Measurement of arterial partial pressure of CO_2_ (PaCO_2_), and not end-tidal CO_2_, (as it may grossly underestimate PaCO_2_ when physiological dead space is increased), is needed to determine the relative contribution of each to high *V*_E_/*V*CO_2_. As repeatedly highlighted in this series, *V*_E_/*V*CO_2_ must be considered in the context of the prevailing mechanical constraints, which can blunt the normal ventilatory response and compound interpretation. For simplicity, measurement of *V*_E_/*V*CO_2_ *nadir* is the preferred parameter: it generally occurs at relatively low exercise intensities, when arterial O_2_ saturation is normal and before the onset of both metabolic acidosis (with ventilatory compensation) and significant restrictive mechanical constraints on tidal volume (V_T_) expansion (O'Donnell et al., 2017, 2019; Neder et al., 2019).

Recent refinements in the “non-invasive” assessment of dynamic respiratory mechanics during exercise have increased our ability to ascertain—with reasonable precision—the nature and severity of the physiological impairment in the symptomatic individual (Milne et al.). The combination of operating lung volumes [measured by serial inspiratory capacity (IC) maneuvers] and breathing pattern can help detect important inspiratory mechanical constraints, which are relevant to dyspnea and exercise limitation (Laveneziana et al., 2009, 2011, 2013a,b, 2014, 2015; Laviolette and Laveneziana, 2014; O'Donnell et al., 2017, 2019; Neder et al., 2019; Boucly et al., 2020). This approach is arguably more sensitive than traditional assessments of breathing reserve (*V*_E_/derived maximal ventilatory capacity), especially in milder forms of obstructive and restrictive disorders or other cardio-respiratory conditions (Laveneziana et al., 2009, 2011, 2013a,b, 2014, 2015; Boucly et al., 2020). Additionally, examination of tidal vs. maximal flow-volume loops throughout exercise provides qualitative assessments of inspiratory and expiratory flow reserves. For practical purposes, the IC, and not the vital capacity, represents the true operating limits for V_T_ expansion in older individuals with common respiratory conditions, especially if expiratory flow limitation is present. Resting IC is variably reduced in both obstructive and restrictive lung disorders (James et al.; Molgat-Seon et al.). The lower the resting IC, the closer V_T_ is positioned to total lung capacity (TLC) and to the upper extremity of the respiratory system's sigmoidal pressure-volume relation. Thus, as V_T_ expands to occupy ~70% of IC and inspiratory reserve volume (IRV) reaches its minimal value, higher IND and breathing effort is needed to achieve a given V_T_. This reflects the increased elastance of the respiratory system, when breathing close to TLC. Appearance of the V_T_ inflection or plateau (minimal IRV), as a function of increasing *V*_E_ during exercise, is an important mechanical event and heralds the onset of NMD and an abrupt rise in dyspnea. As resting IC diminishes over time in obstructive and restrictive disorders, critical inspiratory mechanical constraints and onset of dyspnea will occur at progressively lower V_E_ during exercise (O'Donnell et al., 2017, 2019; Neder et al., 2019).

This series has provided a comprehensive physiological characterization of patients with COPD, ILD, and PAH, (James et al.; Molgat-Seon et al.; Laveneziana and Weatherald) and taught us that exercise response pattern abnormalities are surprisingly similar, despite vastly different pathological origins. For patients referred with unexplained exertional dyspnea, who do not manifest any of the described abnormal patterns during CPET, alternative diagnoses such as dysfunctional breathing of psychogenic origin should be considered (Ionescu et al.). We hope that the pragmatic approach to CPET proposed here will help the clinician to individualize management plans by providing clear answers to a few key questions: *how severe is dyspnea and exercise intolerance in my patient*?; *is ventilatory demand increased and if so, why?*; and *are dynamic mechanical abnormalities present that contribute to poor exercise performance*?

## Author Contributions

DEO wrote the first draft of the manuscript. All authors were responsible for critically reviewing the manuscript for intellectual content and approved the final version of the manuscript submitted for publication.

## Conflict of Interest

The authors declare that the research was conducted in the absence of any commercial or financial relationships that could be construed as a potential conflict of interest.

## References

[B1] Boucly A. Morélot-Panzini C. Garcia G. Weatherald J. Jaïs X. Savale L.. (2020). Intensity and quality of exertional dyspnoea in patients with stable pulmonary hypertension. Eur. Respir. J. 55:1802108. 10.1183/13993003.02108-2018 31771998

[B2] Jones N. L. (1997). Clinical Exercise Testing. Philadelphia, PA: WB Saunders.

[B3] Jones N. L. Killian K. J. (2000). Exercise limitations in health and disease. N. Engl. J. Med. 343, 632–549. 10.1056/NEJM200008313430907 10965011

[B4] Killian K. J. Jones N. L. (1984). The use of exercise testing and other methods in the investigation of dyspnea. Clin. Chest Med. 5, 99–108. 10.1016/S0272-5231(21)00235-5 6723247

[B5] Laveneziana P. Bruni G. I. Presi I. Stendardi L. Duranti R. Scano G. (2013a). Tidal volume inflection and its sensory consequences during exercise in patients with stable asthma. Respir. Physiol. Neurobiol. 185, 374–379. 10.1016/j.resp.2012.08.026 23026436

[B6] Laveneziana P. Garcia G. Joureau B. Nicolas-Jilwan F. Brahimi T. Laviolette L.. (2013b). Dynamic respiratory mechanics and exertional dyspnoea in pulmonary arterial hypertension. Eur. Respir. J. 41, 578–587. 10.1183/09031936.00223611 22790921

[B7] Laveneziana P. Humbert M. Godinas L. Joureau B. Malrin R. Straus C.. (2015). Inspiratory muscle function, dynamic hyperinflation and exertional dyspnoea in pulmonary arterial hypertension. Eur. Respir. J. 45, 1495–1498. 10.1183/09031936.00153214 25931490

[B8] Laveneziana P. Montani D. Dorfmüller P. Girerd B. Sitbon O. Jaïs X.. (2014). Mechanisms of exertional dyspnoea in pulmonary veno-occlusive disease with EIF2AK4 mutations. Eur. Respir. J. 44, 1069–1072. 10.1183/09031936.00088914 25142489

[B9] Laveneziana P. O'Donnell D. E. Ofir D. Agostoni P. Padeletti L. Ricciardi G.. (2009). Effect of biventricular pacing on ventilatory and perceptual responses to exercise in patients with stable chronic heart failure. J. Appl. Physiol. 106, 1574–1583. 10.1152/japplphysiol.90744.2008 19246658

[B10] Laveneziana P. Webb K. A. Ora J. Wadell K. O'Donnell D. E. (2011). Evolution of dyspnea during exercise in COPD: Impact of critical volume constraints. Am. J. Respir. Crit. Care Med. 184, 1367–1373. 10.1164/rccm.201106-1128OC 21885624

[B11] Laviolette L. Laveneziana P. (2014). Dyspnoea: a muldimensional and multidisciplinary approach. Eur. Respir. J. 43, 1750–1762. 10.1183/09031936.00092613 24525437

[B12] Neder J. A. Berton D. C. Marillier M. Bernard A. C. O'Donnell D. E. (2019). Inspiratory constraints and ventilatory inefficiency are superior to breathing reserve in the assessment of exertional dyspnea in COPD. COPD 16, 174–181. 10.1080/15412555.2019.1631776 31272243

[B13] O'Donnell D. E. Elbehairy A. F. Berton D. C. Domnik N. J. Neder J. A. (2017). Advances in the evaluation of respiratory pathophysiology during exercise in chronic lung diseases. Front. Physiol. 8:82. 10.3389/fphys.2017.00082 28275353 PMC5319975

[B14] O'Donnell D. E. Milne K. M. Vincent S. G. Neder J. A. (2019). Unraveling the causes of unexplained dyspnea: the value of exercise testing. Clin. Chest Med. 40, 471–499. 10.1016/j.ccm.2019.02.014 31078223

[B15] Wasserman K. Hansen J. E. Sue D. Y. Stringer W. W. (2012). Principles of Exercise Testing and Interpretation: Including Pathophysiology and Clinical Applications. Philadelphia, PA: Lippincott Williams and Wilkins.

[B16] Wasserman K. Hansen J. E. Sue D. Y. Stringer W. W. Sietsema K. E. Sun X. G.. (1999). Principles of Exercise Testing and Interpretation. Baltimore, MD: Lippincott Williams and Wilkins.

